# RABV induces biphasic actin cytoskeletal rearrangement through Rac1 activity modulation

**DOI:** 10.1128/jvi.00606-24

**Published:** 2024-05-29

**Authors:** Xiaomin Liu, Jing Xu, Maolin Zhang, Hualei Wang, Xin Guo, Mingxin Zhao, Ming Duan, Zhenhong Guan, Yidi Guo

**Affiliations:** 1 Institute of Zoonosis, College of Veterinary Medicine, Jilin University, State Key Laboratory for Diagnosis and Treatment of Severe Zoonotic Infectious Diseases, Key Laboratory for Zoonosis Research of the Ministry of Education, Changchun, China; St. Jude Children's Research Hospital, Memphis, Tennessee, USA

**Keywords:** rabies virus, Rac1, phosphoprotein, actin rearrangement, signal transduction

## Abstract

Rabies virus (RABV) is highly lethal and triggers severe neurological symptoms. The neuropathogenic mechanism remains poorly understood. Ras-related C3 botulinum toxin substrate 1 (Rac1) is a Rho-GTPase that is involved in actin remodeling and has been reported to be closely associated with neuronal dysfunction. In this study, by means of a combination of pharmacological inhibitors, small interfering RNA, and specific dominant-negatives, we characterize the crucial roles of dynamic actin and the regulatory function of Rac1 in RABV infection, dominantly in the viral entry phase. The data show that the RABV phosphoprotein interacts with Rac1. RABV phosphoprotein suppress Rac1 activity and impedes downstream Pak1-Limk1-Cofilin1 signaling, leading to the disruption of F-actin-based structure formation. In early viral infection, the EGFR-Rac1-signaling pathway undergoes a biphasic change, which is first upregulated and subsequently downregulated, corresponding to the RABV entry-induced remodeling pattern of F-actin. Taken together, our findings demonstrate for the first time the role played by the Rac1 signaling pathway in RABV infection and may provide a clue for an explanation for the etiology of rabies neurological pathogenesis.

**IMPORTANCE:**

Though neuronal dysfunction is predominant in fatal rabies, the detailed mechanism by which rabies virus (RABV) infection causes neurological symptoms remains in question. The actin cytoskeleton is involved in numerous viruses infection and plays a crucial role in maintaining neurological function. The cytoskeletal disruption is closely associated with abnormal nervous symptoms and induces neurogenic diseases. In this study, we show that RABV infection led to the rearrangement of the cytoskeleton as well as the biphasic kinetics of the Rac1 signal transduction. These results help elucidate the mechanism that causes the aberrant neuronal processes by RABV infection and may shed light on therapeutic development aimed at ameliorating neurological disorders.

## INTRODUCTION

Rabies virus (RABV), a negative-sense RNA virus belonging to the genus *Lyssavirus* family *Rhabdoviridae*, is neurotropic and the causative agent of rabies (1). RABV in the saliva of an infected animal enters peripheral nerves from a bite site, primarily migrates retrogradely by neural axons, and eventually reaches the central nervous system (CNS) (2). A range of neurological symptoms strikes, including severe agitation, depression, hydrophobia, paralysis, and impaired consciousness, and ultimately ends with coma and death (3). Interestingly, neuronal loss remains minimal, while neuronal dysfunction is predominant (4, 5). Pathogenic RABV infection leads to severe neuronal process destruction, possibly induced by the breach and discontinuity of the cytoskeleton network (microtubules and filamentous actin) (6, 7). RABV interacts with the cell actin cytoskeleton in a number of ways (8, 9). RABV has been reported to cause the depolymerization of neuronal filamentous actin and dendritic injuries *in vivo* and *in vitro* (10–12). A challenge virus strain (CVS) of RABV influences more than 90% of cytoskeleton-related gene expression (13). Nevertheless, details on the roles played by the actin cytoskeleton and related mechanisms, especially the key regulators involved, remain to be explored.

Facilitated by accessory factors, the monomeric form of globular actin (G-actin) polymerizes to constitute filamentous actin (F-actin) (14). Disruption of the actin network leads to neurodegenerative diseases (7). The actin dynamic equilibrium between assembly and disassembly is manipulated by signaling molecules, such as the Rho family of GTPases (15). Ras-related C3 botulinum toxin substrate 1 (Rac1), Transforming protein RhoA (RhoA), and Cell division control protein 42 homolog (Cdc42) were the first-identified and best-characterized members of the Rho-GTPase family (16). Small GTPases switch between inactive (GDP-bound) and active (GTP-bound) forms and elicit various biological functions through multiple downstream signaling pathways, including gene expression, cell migration, and actin cytoskeleton rearrangement (17, 18). Among these, Rac1 has been reported to facilitate viral endocytosis and invasion of various viruses, such as porcine reproductive and respiratory syndrome virus, hepatitis B virus, herpes simplex virus-1 (HSV-1), African swine fever virus (ASFV), and influenza A virus (IAV) (19–23). The involvement of Rac1 in RABV infection is unclear, and the association between Rac1-regulated actin rearrangement and RABV-induced neuronal dysfunction also awaits clarification. Thus, exploring the actin remodeling pattern and the underlying mechanisms that are stimulated by RABV infection is of great importance.

In the present study, we demonstrate that the homeostasis of dynamic actin sustains RABV infection with the involvement of the Rac1 signaling pathway. The data show that Rac1 activity affects RABV infection by regulating dynamic actin primarily at the viral entry stage. RABV suppresses Rac1 activity through the interaction of RABV phosphoprotein (P) and Rac1, eventually leading to F-actin depolymerization. Additionally, RABV induces biphasic dynamics of epidermal growth factor receptor (EGFR)-phosphatidylinositide-3 kinase (PI3K)-Rac1 signaling at early infection and impedes Rac1-p21-activated kinase 1 (Pak1) transduction later. These indicate that actin assembly facilitates viral entry, and subsequently, F-actin tends to develop a form of disorder in later stages of infection. Taken together, these data reveal, for the first time, the crucial roles played by Rac1 in the regulation of RABV infection as well as in mediating RABV-induced actin rearrangement.

## RESULTS

### RABV infection requires a dynamic actin cytoskeleton

**Fig 1:**
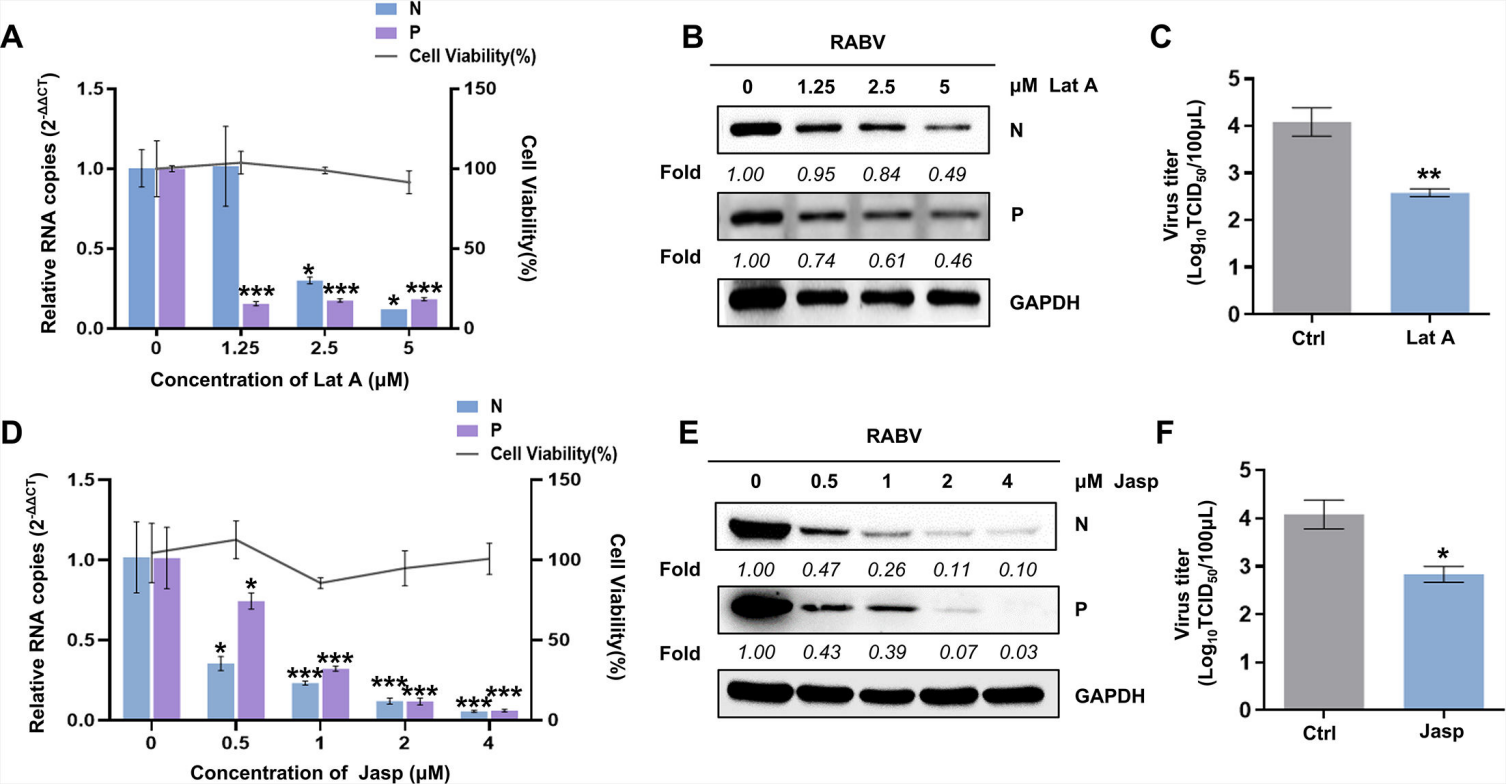
Disruption of actin dynamics impairs RABV infection. (**A–F**) Following MTT assay performed to exclude cytotoxic side effects upon drugs treatment, N2a cells were pretreated with increasing concentrations of Latrunculin A (Lat A; 0, 1.25, 2.5, and 5 µM) (**A**) or Jasplakinolide (Jasp; 0, 0.5, 1, 2, and 4 µM) (**D**) for 1 h and then infected with CVS-11 (MOI = 1). At 24 h p.i., cells were lysed to determine the RNA copy numbers of RABV N and P by real-time qPCR (RT-qPCR) (A and D). The protein levels of RABV N and P were measured by western blot and analyzed by densitometric analysis using ImageJ normalized to GAPDH (B and E). With pretreatment of Lat A (5 µM) (**C**) or Jasp (4 µM) (**F**), the viral titer in the cell culture supernatant was quantified by TCID50 assay at 24 h p.i. Results are represented as mean ± SD of three independent experiments. Statistical significances of the differences are indicated. Student’s *t* test, *P* < 0.05 (*); *P* < 0.01 (**); *P* < 0.001 (***).

To examine whether RABV infection relies on the actin cytoskeleton, Latrunculin A (Lat A, an actin polymerization inhibitor) and Jasplakinolide (Jasp, an actin-stabilizing drug) were used in experiments (24, 25). Methylthiazolyldiphenyl-tetrazolium bromide (MTT) assays were first performed to exclude cytotoxic side effects upon drug treatment. Neuro-2a cells (N2a) were pretreated with increasing concentrations of Lat A (0, 1.25, 2.5, and 5 µM) or Jasp (0, 0.5, 1, 2, and 4 µM) for 1 h and infected with CVS-11 at a multiplicity of infection (MOI) of 1. mRNA and whole-cell protein were harvested at 24 h postinfection (p.i.). The results showed that the RNA and protein levels of RABV N and P were significantly decreased in a dose-dependent manner (Fig. 1A and B). The viral titer was reduced following Lat A pretreatment (5 µM), as detected by 50% tissue culture infectious dose (TCID50) assay (Fig. 1C). Simultaneously, Jasp showed a similar inhibitory effect on RABV infection, which intensified with increasing amounts of the drug (Fig. 1D and E). Accordingly, the viral titer was attenuated significantly by 30% (Fig. 1F). Taken together, chemical inhibitors of actin polymerization and depolymerization both suppressed viral proliferation, which indicates that an intact actin network is critical for RABV infection.

### Rac1 activity is involved in the regulation of RABV infection

**Fig 2:**
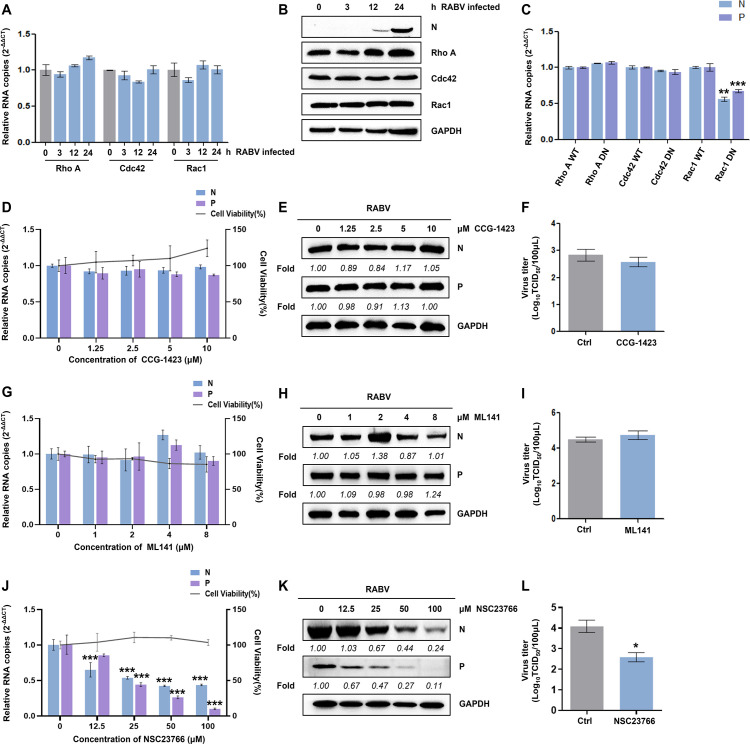
Rho GTPase Rac1 is involved in RABV infection. (A and B) N2a cells were inoculated with CVS-11 (MOI = 1) and harvested at 0, 3, 12, and 24 h p.i.; the mRNA and protein levels of RhoA, Cdc42, and Rac1 were detected by RT-qPCR (**A**) and western blot (**B**). (**C**) N2a cells were transfected with WT or the DN mutant forms of RhoA, Rac1, or Cdc42 for 24 h. After being infected with CVS-11 (MOI = 1) for 24 h, cells were harvested for mRNA quantification of RABV N and P. (**D–L**) Following ruling out the toxic effects of drugs, CCG-1423, ML141, and NSC23766, by MTT assays, N2a cells were disposed with increasing concentrations of CCG-1423 (0, 1.25, 2.5, 5, and 10 µM) (**D**), ML141 (0, 1, 2, 4, and 8 µM) (**G**), or NSC23766 (0, 12.5, 25, 50, and 100 µM) (**J**) for 1 h and then infected with CVS-11 (MOI = 1) for 24 h p.i. Cells were collected to detect the RNA copy numbers of RABV N and P by RT-qPCR. The protein levels of RABV N and P were examined by western blot and analyzed by densitometric analysis using ImageJ normalized to GAPDH (E, H, and K). With treatment of CCG-1423 (10 µM) (**F**), ML141 (8 µM) (**I**), or NSC23766 (100 µM) (**L**), the viral titer in the cell culture supernatant was quantified by TCID50 assay at 24 h p.i. Results are represented as mean ± SD of three independent experiments. Statistical significances of the differences are indicated. Student’s *t* test, *P* < 0.05 (*); *P* < 0.01 (**); *P* < 0.001 (***).

RhoA, Cdc42, and Rac1, members of the Rho-GTPase family, regulate actin cytoskeleton rearrangements. The endogenous levels of Rac1, RhoA, and Cdc42 were initially measured, and no significant change was observed during RABV replication (Fig. 2A and B). To investigate the roles of the above Rho-GTPases in RABV infection, N2a cells were transfected with wild-type or dominant-negative mutant of RhoA, Cdc42, and Rac1, followed by CVS-11 inoculation at an MOI of 1 for 24 h. The transfection efficiency reached over 70%. Negative dominant Rac1 exerted an inhibitory effect on the RNA copy number of RABV N and P, while RhoA and Cdc42 did not affect RABV protein expression (Fig. 2C). We employed inhibitor treatment to verify the involvement of these three GTPases in RABV infection after evaluation of the optimal drug concentration. RhoA inhibitor CCG-1423 and Cdc42 inhibitor ML141 both had no obvious impact on RABV replication, as observed by viral protein expression level and virus titer (Fig. 2D through I). However, as the NSC23766 concentration increased from 0 to 100 µM, viral N and P RNA copy numbers were reduced in a dose-dependent manner (Fig. 2J). The protein expression of RABV N and P showed a similar trend (Fig. 2K). Moreover, TCID50 data showed that the viral titer declined to 50% after NSC23766 treatment (Fig. 2L). These data indicate that Rac1, but not RhoA or Cdc42, positively regulates RABV infection.

**Fig 3:**
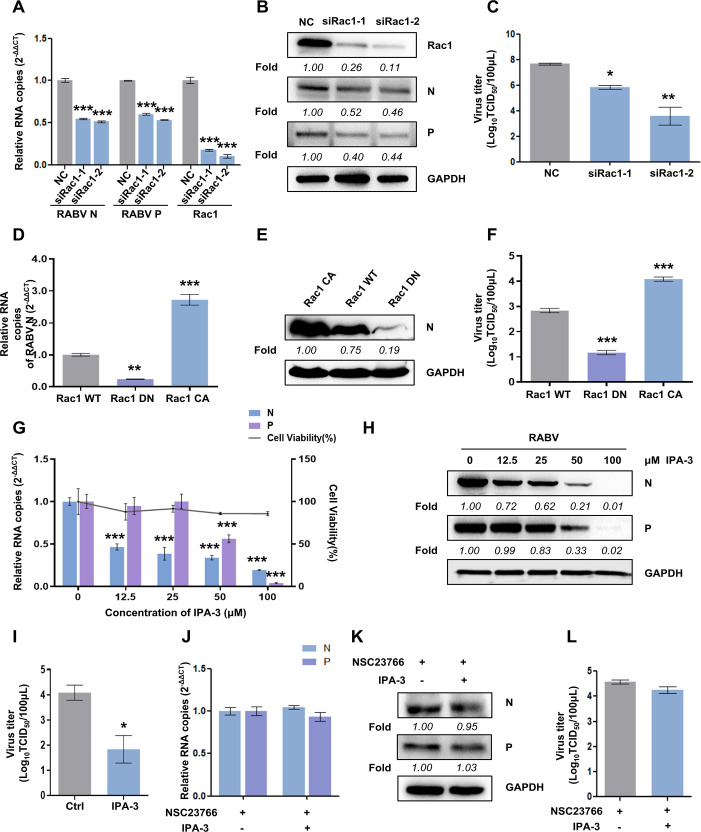
Perturbation of Rac1 activity interferes with RABV infection. (**A–C**) N2a cells were transfected with Rac1-siRNA-1, Rac1-siRNA-2, and nonspecific control siRNA (NC), respectively, followed by infection with CVS-11 at MOI of 1 at 24 h post-transfection. At 24 h p.i., the cells were lysed to determine the RNA copy numbers of RABV N, P, and Rac1 (**A**). (**B**) The protein levels of RABV N, P, and Rac1 were determined by western blot and quantitated by densitometric analysis using ImageJ. GAPDH was used as a loading control. (**C**) Cell culture supernatant was harvested at 24 h p.i., and virus titer was measured by TCID50. (**D–F**) N2a cells were transfected with either constitutively active Rac1 (Rac1 CA) or dominant-negative Rac1 (Rac1 DN) or wild-type (Rac1 WT) forms followed by CVS-11 infection (MOI = 1) at 24 h posttransfection. At 24 h p.i., relative RNA copies and protein level of RABV N were examined by RT-qPCR (**D**) and western blot (**E**), respectively, normalized to that of GAPDH. (**F**) TCID50 assay was also performed to quantify the viral titer. (**G–I**) N2a cells were pretreated with the indicated concentration of IPA-3 (0, 12.5, 25, 50, and 100 µM) for 1 h and subsequently infected with CVS-11 (MOI = 1). Cytotoxicity of IPA-3 on N2a cells was analyzed by MTT, and RNA copy numbers of RABV (N and P) were detected by RT-qPCR (**G**). The protein levels of RABV N and P were measured by western blot and were quantified by densitometric analysis using ImageJ, with GAPDH used as a loading control (**H**). (**I**) The viral titer was quantified by TCID50 assay with pretreatment of IPA-3 at 100 µM. (**J–L**) Cells were pretreated with NSC23766, IPA-3, or both, respectively, for 1 h and infected with CVS-11 at MOI of 1. At 24 h p.i., N2a cells were harvested and lysed. The expression levels of RABV N and P were determined by RT-qPCR (**J**) and western blot (**K**). GAPDH was used as a loading control. (**L**) The viral titer in the cell culture supernatant was also measured at 24 h p.i. Results are represented as mean ± SD of three independent experiments. Statistical significances of the differences are indicated. Student’s *t* test, *P* < 0.05 (*); *P* < 0.01 (**); *P* < 0.001 (***).

To further clarify the role of Rac1 during RABV replication, we knocked down Rac1 via RNA interference and then inoculated cells with CVS-11 (MOI = 1) for 24 h. Two short-interfering RNA oligonucleotides (siRNA-1 and siRNA-2) specifically targeting Rac1 were employed to minimize possible off-target effects, with small interfering RNA (siRNA)-negative control (NC) duplexes used as a control. The RT-qPCR data showed that the RNA level of RABV N and P were both decreased by almost one-half, while efficient downregulation of Rac1 was achieved at approximately 90% (Fig. 3A). Silencing Rac1 suppressed the expression level of RABV N and P, which was determined by western blot and was analyzed by the band density (Fig. 3B). Consistently, the viral titer was reduced (Fig. 3C). We expressed Rac1 mutant forms prior to infection and obtained comparable results. The constitutively active Rac1 mutant significantly increased the expression of RABV N (Fig. 3D and E) as well as the viral titer (Fig. 3F), indicating a significant promotion in RABV infectivity. In contrast, inactive Rac1 impeded the viral infection level compared with the effect of Rac1 WT, which was consistent with the outcome of NSC23766 treatment. As Paks are serine/threonine kinases downstream of the Rac1 signaling pathway, we pretreated N2a cells with different concentrations of IPA-3, a direct, noncompetitive, and highly selective Pak1-3 inhibitor. RT-qPCR and western blot showed that RABV N and P expressions were reduced in a dose-dependent manner (Fig. 3G and H), and TCID50 data showed that the viral titer dropped significantly (Fig. 3I). When cells were simultaneously treated with NSC23766 and IPA-3, the inhibitory impact of IPA-3 on viral infection was nearly abolished compared with NSC23766 treated group according to Fig. 3J through L. In summary, our results implicate that Rac1 activity is involved in RABV infection regulation through downstream factors Paks.

### RABV P interacts with cellular Rac1

**Fig 4:**
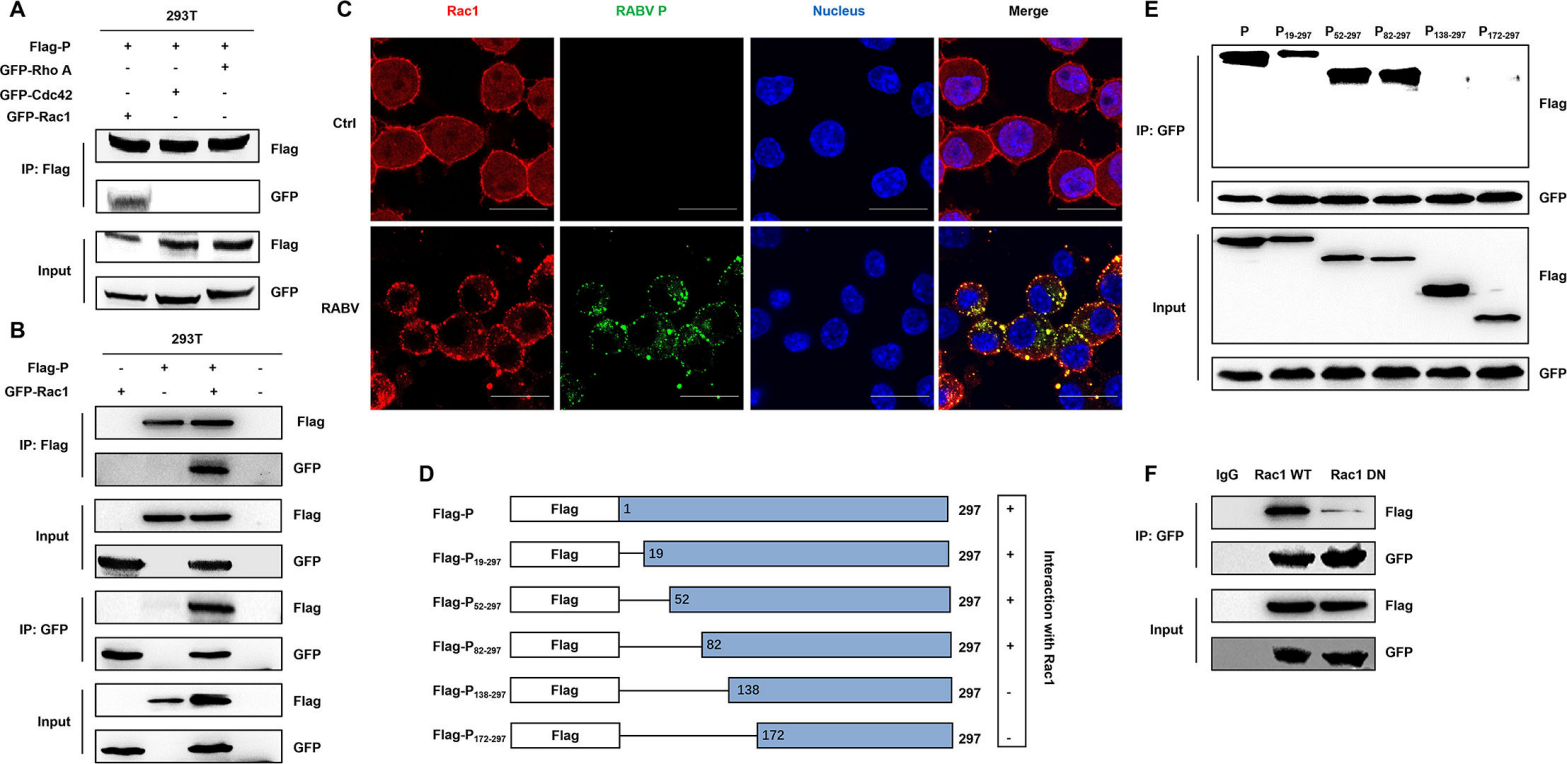
RABV P interacts with Rac1. (**A**) Cotransfection of Flag-tagged P and GFP-tagged RhoA, Cdc42, or Rac1 was conducted in 293T cells, and co-IP assay was employed with lysates harvested from 293T cells using anti-Flag antibody (Flag) to identify the interaction between pairwise pairs as indicated. (**B**) Co-IP was performed with lysates harvested from 293T cells with Flag-tagged P, GFP-tagged Rac1, or both expressed as indicated. Proteins were precipitated with anti-Flag antibody (Flag) and anti-GFP antibody (GFP) and examined by western blot. (**C**) N2a cells were infected with CVS-11 (MOI = 1) and fixed at 48 h p.i. The RABV P (green) and endogenous Rac1 (red) were detected by immunofluorescence staining under a confocal microscope. The nuclei were stained with Hochest (blue). Bars, 20 µm. (**D**) The map showed the structure of full-length P and its truncated forms employed in this study, including Flag-fused constructs (Flag-P, Flag-P_19-297_, Flag-P_52-297_, Flag-P_82-297_, Flag-P_138-297_, and Flag-P_172-297_). (**E**) Flag-fused P constructs (Flag-P, Flag-P_19-297_, Flag-P_52-297_, Flag-P_82-297_, Flag-P_138-297_, and Flag-P_172-297_) were, respectively, co-transfected with GFP-Rac1 into 293T cells. At 48 h posttransfection, co-IP and western blot were performed with anti-Flag and anti-GFP antibody. (**F**) 293T cells were lysed after Flag-fused P full-length cotransfection with Rac1 WT or Rac1 DN for 48 h. Co-IP was performed with proteins precipitated with anti-Flag and anti-GFP antibody in western blot.

Given the intricate function characterized by RABV P, we constructed a Flag-conjugated plasmid encoding RABV P. Through immunoprecipitation-mass spectrometry (IP-MS) data, we found that Rac1 was among the potential host factors associated with RABV P (Tables S1 and S2). After transfection of Flag-P with GFP-Rac1, GFP-Cdc42, or GFP-RhoA, respectively, into 293T cells, coimmunoprecipitation (co-IP) was performed. Flag-P was immunoprecipitated with GFP-Rac1, but not with GFP-Cdc42 or GFP-RhoA (Fig. 4A). As shown in Fig. 4B, co-IP using anti-Flag and reverse co-IP experiments using anti-GFP validated that P interacted with Rac1. After N2a cells were inoculated with CVS-11 at an MOI of 1 for 48 h, we performed an immunoﬂuorescence assay and observed the colocalization of RABV P and endogenous Rac1 (Fig. 4C). To determine the interaction sequence of RABV P with Rac1, a series of Flag-tagged truncated P proteins were constructed, namely, P_19-297_ (consisting of amino acids 19–297), P_52-297_ (consisting of amino acids 52–297), P_82-297_ (consisting of amino acids 82–297), P _138-297_ (consisting of amino acids 138–297), and P_172-297_ (consisting of amino acids 172–297; Fig. 4D), and cotransfected them, respectively, with GFP-Rac1 into 293T cells. According to the co-IP data, P, P_19-297_, P_52-297_, and P_82-297_ bound Rac1 (lanes 1, 2, 3, and 4), while deletion mutants with the removal of the residues between 1–137 and 1–171 completely abrogated the association of the P protein and Rac1 (lanes 5 and 6; Fig. 4E). To identify the interaction sites of Rac1, we cotransfected Flag-P with GFP-WT Rac1 or GFP-Rac1 DN (T17N) in 293T cells. In Fig. 4F, the amount of RABV P coprecipitated with GFP-Rac1 DN was markedly decreased compared with those coprecipitated with GFP-Rac1 WT. Collectively, these data reveal that RABV P interacts with Rac1 through the 82–137 amino acid region, and the 17th amino site in Rac1 possibly played a crucial role.

### RABV P suppresses Rac1 activity

**Fig 5:**
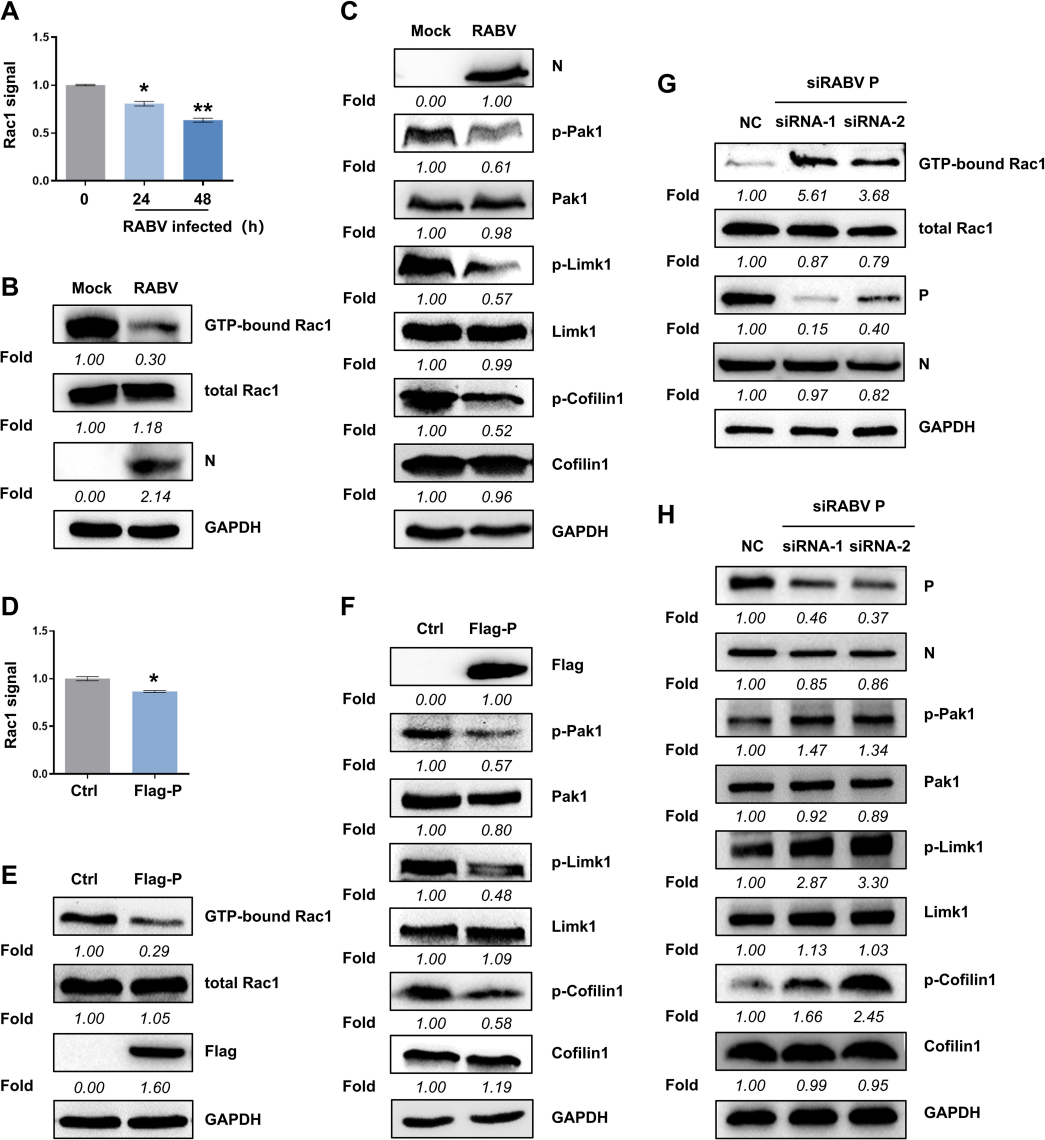
RABV P downregulates Rac1 activity. (**A–C**) N2a cells were infected by CVS-11 at MOI of 1. At 24 and 48 h p.i., total cell lysis was collected. Rac1 activity was measured by G-lisa assay (**A**). GTP-bound Rac1 was precipitated using Pak1 PBD-Agarose Beads in pull-down assay and processed for western blot analysis. The density of GTP-bound Rac1, total Rac1, and RABV N was quantified compared with GAPDH by ImageJ (**B**). The phosphorylation and total protein level of Rac1 signaling downstream molecules (Pak1, Limk1, and Cofilin1) were detected by western blot (**C**). (**D–F**) Flag-P was transfected into N2a cells for 48 h as an empty vector used as a control. Total cell lysis was collected. G-lisa assay was performed to measure Rac1 activity (**D**), and a pull-down assay was performed to analyze the amount of GTP-bound Rac1 compared with that of total Rac1 (**E**). The p-Pak1, p-Limk1, and p-Cofilin1 were immunoblotted and analyzed by the mean density compared with total amounts of Pak1, Limk1, and Cofilin1, respectively (**F**). Flag expression was measured as a control of transfection. (G and H) After being inoculated with CVS-11 (MOI = 0.1) for 24 h, N2a cells were transfected with RABV P-siRNA-1, RABV P-siRNA-2, and nonspecific control siRNA (NC), respectively, for 24 h, and total protein was collected from the lysed cells at 24 h p.i. Glutathione S-transferase (GST) pull-down assay was proceeded by contrasting the amount of GTP-bound Rac1 to total Rac1 (**G**), and the phosphorylation and total protein level of Pak1, Limk1, and Cofilin1 were determined by western blot (**H**). GAPDH was used as a loading control. Results are represented as mean ± SD of three independent experiments. Statistical significances of the differences are indicated. Student’s *t* test, *P* < 0.05 (*); *P* < 0.01 (**); *P* < 0.001 (***).

Because RABV infection can be regulated by Rac1 activity and since RABV P binds to Rac1, we hypothesized that RABV may affect Rac1 GTPase activity. To test this hypothesis, we employed G-lisa and pull-down techniques to analyze Rac1 activity in CVS-11-infected and P-overexpressed N2a cells. As Rac1 function is regulated via a switch between an inactive GDP-bound state and an active GTP-bound state, the GTP level was measured. The activity of endogenous Rac1 was significantly and continuously reduced, dropping to three-quarters that of the mock-infected group at 48 h p.i. (Fig. 5A). A western blot analysis showed that GTP-bound Rac1 levels declined by 70% when normalized to endogenous total Rac1 (Fig. 5B). Simultaneously, the phosphorylation of Rac1 downstream factors, Pak1, Limk1, and Cofilin1, were all suppressed, while the total protein level remained constant (Fig. 5C). Accordingly, in transiently P-overexpressing N2a cells, Rac1 activity was dampened and was approximately 80% of that of the empty vector-transfected in G-lisa (Fig. 5D) and 30% in the pull-down assay (Fig. 5E). The amounts of p-Pak1, p-Limk1, and p-Cofilin1 were thus decreased (Fig. 5F). Moreover, two siRNA constructs targeting RABV P were synthesized and transfected into cells which were infected with RABV for 24 hours to assess the impact of P knockdown on virus-induced Rac1 activity. The levels of GTP-bound Rac1 were upregulated, and the phosphorylation of Pak1, Limk1, and Cofilin1 was enhanced (Fig. 5G and H), indicating that P knockdown post RABV infection abolished suppression of Rac1 activity by RABV. These data suggest that RABV P downregulates Rac1 activity and blocks downstream signal transduction.

### RABV infection induces actin cytoskeletal remodeling

**Fig 6:**
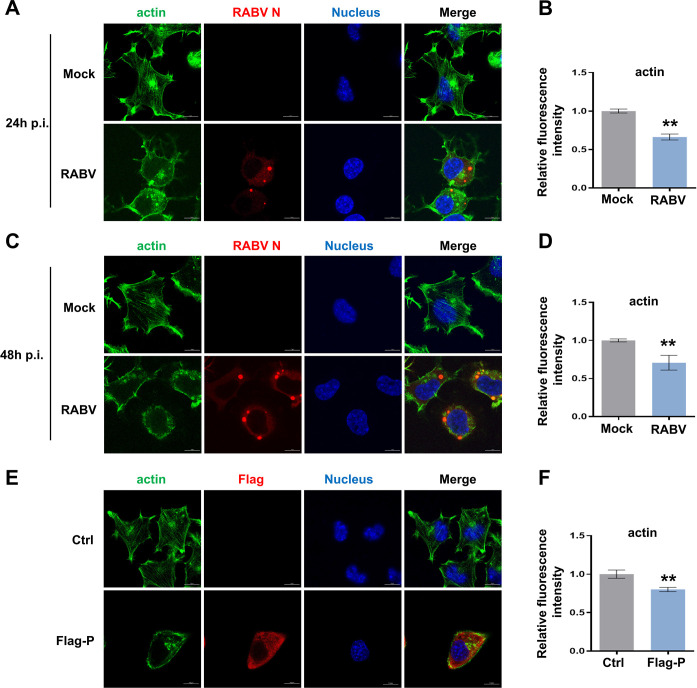
RABV infection leads to actin disassembly. (**A–D**) N2a cells were inoculated with CVS-11 or mock at MOI of 1. Cells were fixed at 24 h (A and B) and 48 h (C and D) p.i. Actin filaments (green) were stained with Alexa Fluor 488-phalloidin, and viral particles (red) were dyed with FITC-anti-Rabies Monoclonal antibody, with nuclei (blue) stained by Hochest. (E and F) Flag-P was transfected into N2a cells for 48 h as an empty vector used as a control. Fixed cells were stained with Alexa Fluor 488-phalloidin, FITC-anti-Rabies Monoclonal antibody, and Hochest to visualize actin filaments (green), viral particles (red), and cell nuclei (blue), respectively. Images were observed and captured by confocal microscopy (**E**). The relative fluorescence intensity of actin was analyzed by ImageJ (**F**). Bars, 10 µm. Results are represented as mean ± SD of three independent experiments. Statistical significances of the differences are indicated. Student’s *t* test, *P* < 0.05 (*); *P* < 0.01 (**); *P* < 0.001 (***).

Rac1 and its downstream kinases are key regulators of cytoskeleton dynamics. To ascertain the influence of RABV infection on the morphological changes in the actin cytoskeleton, actin was stained with Alexa Fluor 488-phalloidin and observed by confocal microscopy. We observed that the disassembly of stress fibers of the actin cytoskeleton was evident in CVS-11-infected cells at both 24 h p.i. and 48 h p.i., contrasting with the mock-infected cells (Fig. 6A and C). The dissolution of actin stress fibers was induced by RABV with increasing time of virus inoculation, which was also analyzed by the fluorescence intensity (Fig. 6B and D). The same phenomenon was also detected when Flag-P was overexpressed in N2a cells (Fig. 6E and F). These results indicate that RABV infection induces actin cytoskeletal disassembly through RABV P inactivation of the Rac1-Pak1 pathway.

### RABV reciprocally influences the EGFR-Rac1 pathway during early infection

**Fig 7:**
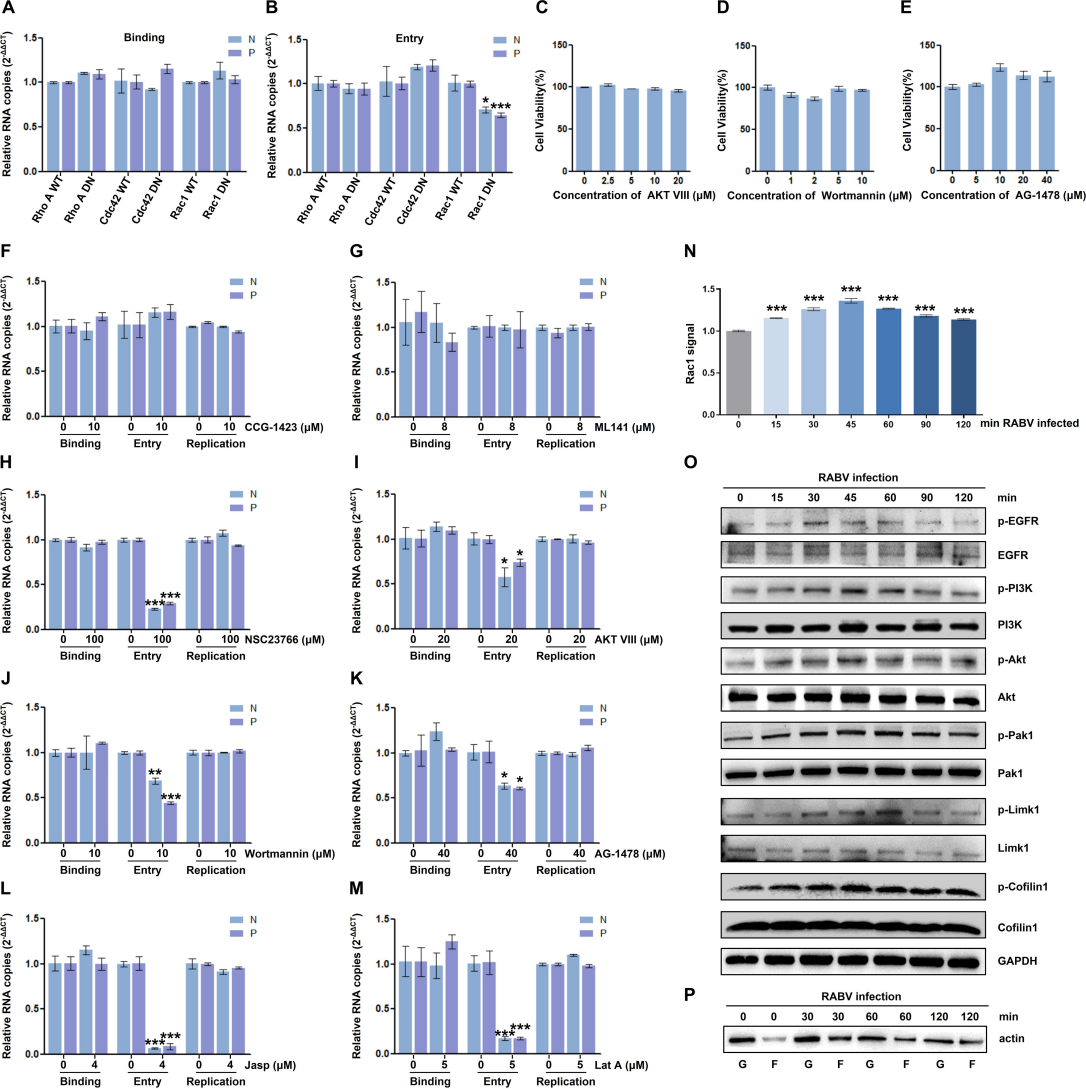
RABV infection is mutually regulated with EGFR-Rac1 in the early stage. (A and B) After transfected with the WT or the DN mutant forms of Rho A, Rac1, or Cdc42 for 24 h, N2a cells were infected with CVS-11 (MOI = 10) for 1 h at 4°C (**A**) and then shifted to 37°C. At 0 h (binding) (**A**) or 2 h (entry) (**B**) p.i., the cell cultures were harvested, and the mRNA levels of RABV N and P were quantified. (**C–E**) N2a cells were, respectively, treated with increasing concentrations of AKT VIII (0, 2.5, 5, 10, and 20 µM) (**C**), Wortmannin (0, 1, 2, 5, and 10 µM) (**D**), or AG-1478 (0, 5, 10, 20, and 40 µM) (**E**). MTT assay was performed to exclude cytotoxic side effects upon drug treatment. (**F–M**) N2a cells were pretreated with indicated concentration of drugs, namely CCG-1423 (10 µM) (**C**), ML141 (8 µM) (**D**), NSC23766 (100 µM) (**E**), AKT VIII (20 µM) (**F**), Wortmannin (10 µM) (**G**), AG-1478 (40 µM) (**H**), Jasp (4 µM) (**I**), and Lat A (5 µM) (**J**), at 37°C for 1 h, inoculated with CVS-11 (MOI  =  10) at 4°C for 1 h, and then shifted to 37°C. At 0 h (binding) or 2 h (entry) p.i., the cell cultures were harvested for RT-qPCR. (**K–M**) N2a cells were inoculated with CVS-11 (MOI = 10) at 4°C for 1 h and then transferred to 37°C for indicated time. Cells were lysed and processed for G-lisa assay to detect Rac1 activity (**K**). The p-EGFR, EGFR, p-PI3K, PI3K, p-Akt, Akt p-Pak1, Pak1, p-Limk1, Limk-1, p-Cofilin1, and Cofilin1 were immunoblotted (**L**). N2a cells were inoculated with CVS-11 (MOI = 10) at 1 h and then transferred to 37°C for 0, 30, 60, and 120 min p.i.. The F-actin and G-actin fractions were prepared and subjected to western blot analysis. G, G-actin from supernatant fraction; F, F-actin from pellet fraction (**M**). Results are represented as mean ± SD of three independent experiments. Statistical significances of the differences are indicated. Student’s *t* test, *P* < 0.05 (*); *P* < 0.01 (**); *P* < 0.001 (***).

Actin is implicated in entry events occurring early in RABV infection. To elucidate the role of Rho GTPases in the early stage of RABV infection, N2a cells were transfected with the WT or the DN mutant forms of RhoA, Rac1, or Cdc42, followed by CVS-11 infection (MOI = 10) for 1 h at 4°C. After shifting to 37°C, the cell cultures were harvested at 0 and 2 h, respectively, to quantify the mRNA copy numbers of RABV N and P for investigating viral binding and entry levels. The RT-qPCR data demonstrated that viral binding remained unaffected by any Rho GTPase, whereas viral entry was specifically inhibited by Rac1 DN rather than RhoA DN or Cdc42 DN (Fig. 7A and B). The inhibitor treatment experiments yielded consistent results. CCG-1423 and ML141 exhibited no impact on both RABV binding and entry (Fig. 7F and G), while NSC23766 influenced viral entry without affecting viral binding (Fig. 7H). EGFR/PI3K/Akt pathway has been reported to induce dynamic changes of actin cytoskeleton, associate with activation of Rho family GTPases, and regulate various viral uptake and propagation (21, 22, 26, 27). To further assess the effect of Rac1 upstream signaling on RABV early infection, we employed EGFR inhibitor AG-1478, PI3K inhibitor Wortmannin, and Akt inhibitor AKT VIII after the optimal drug concentration evaluation (Fig. 7C through E). AG-1478, Wortmannin, and AKT VIII exerted an inhibitory impact on RABV entry but had a negligible effect on viral attachment (Fig. 7I through K). Moreover, Jasp and Lat A pretreatment resulted in a remarkable decrease in RABV internalization, contrasting the unaltered trend observed in viral binding (Fig. 7L and M), which indicated that dynamic actin disorder mainly affects the entry phase of RABV. Additionally, Rac1 activation was investigated at the indicated time points. The level of activated (GTP-bound) Rac1 increased substantially (15–45 min) and subsequently diminished during viral entry (45–120 min; Fig. 7N). Simultaneously, the phosphorylation of EGFR, PI3K, Akt, Pak1, Limk1, and Cofilin1 gradually increased, reaching a maximal level at approximately 30 and 45 min p.i. and showed a decline from 45 to 120 min p.i., while the total protein amount remained unchanged throughout (Fig. 7O). Next, an F-actin/G-actin assay was performed to examine the formation of F-actin at RABV entry. The amount of the polymerized actin (F-actin) level was proportionally upregulated at 30 min p.i. and slightly reduced later (Fig. 7P). In conclusion, EGFR-Rac1 shows dynamic activation during early viral infection and exerts its main regulatory effects upon the RABV entry stage.

## DISCUSSION

**Fig 8:**
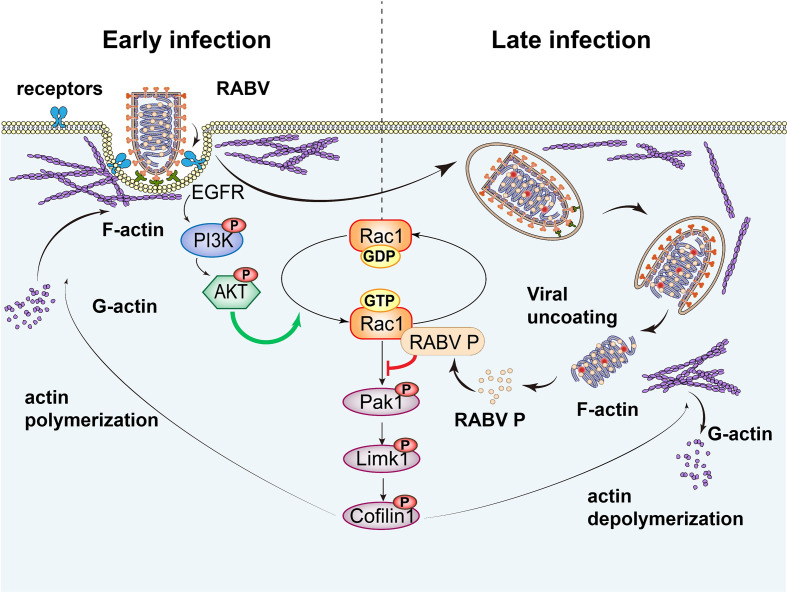
Model of Rac1 regulating actin dynamics during RABV infection. In the early stage of infection, RABV combines with the viral receptor and stimulates the EGFR-Rac1-Pak1 signaling pathway, ultimately promoting F-actin polymerization, which mediates the viral internalization event. In the later stage of infection, subsequent viral uncoating allows the RNP releasement into the cytoplasm. RABV P protein interacts and inactivates Rac1, blocking downstream Pak1-Limk1-Cofilin1 signal transduction, which eventually leads to actin depolymerization. The biphasic activation of Rac1 regulates actin rearrangement and is involved in RABV infection.

In addition to maintaining the integrity, motility, and shape of cells, the actin cytoskeleton is disrupted or taken over by extracellular pathogens (28). Numerous viruses manipulate actin networks and create optimal niches for multiple events in the viral life cycle, including internalization, replication, and spread (14, 29). For example, HIV-1 subverts actin remodeling at the cell surface prior to entry and interacts with the actin cytoskeleton during later stages of infection (30, 31). Herpesviruses usurp the actin cytoskeleton and actin-regulating Rho-GTPase signaling pathways during entry, replication, and egress (32). In this study, pharmacological treatment with Lat A or Jasp inhibited RABV infection primarily at an early stage (Fig. 1 and 7), which indicates that intact actin facilitates viral internalization. RABV has been reported to share a clathrin-mediated and actin-dependent endocytic pathway with other rhabdoviruses, such as vesicular stomatitis virus (33–35). After virion attachment to the cell plasma membrane, actin is polymerized to provide sufficient force to counteract the elevated membrane tension and facilitate viral clathrin-mediated internalization (36). Various cellular signaling pathways are promptly initiated (21, 22, 37–39). However, the kinases and signaling pathways of RABV entry remain poorly understood. We show here that RABV triggers biphasic kinetics of the EGFR-Rac1-Pak1 signal transduction pathway, which is first upregulated and subsequently downregulated (Fig. 7). Meanwhile, the formation of F-actin-based structures is enhanced at an early stage of infection and subsequently weakened (Fig. 7). Rac1 activity acts dominantly in RABV entry but not considerably in attachment or the later infection period, along with upstream factors including EGFR, PI3K, and Akt, which indicated that halting EGFR-PI3K-Akt-Rac1 possibly influenced mostly on viral entry (Fig. 7). EGFR signaling network is widely utilized for viral entry and cytoskeleton remodeling. Hepatitis C virus (HCV) binding activated EGFR, which, in turn, helps the assembly of HCV entry cofactor complex and promotes HCV entry (40). ASFV directly stimulates EGFR, PI3K-Akt, Pak1, and Rac1 activation and regulates its micropinocytosis (22). The study on whether EGFR signaling associates with RABV internalization manner and whether EGFR regulates the interaction of viral protein with the putative RABV receptors is very much expected. A model is proposed that RABV binding stimulates activation of the EGFR-PI3K-Akt-signaling pathway, promoting Rac1-induced F-actin polymerization, thereby facilitating clathrin-mediated RABV endocytosis. In the later stage, following the exposure of RABV ribonucleoprotein complex (RNP) after uncoating, P binds and inactivates Rac1, initiating cascades of phosphorylation of downstream regulators and resulting in actin depolymerization (Fig. 8). Similar biphasic F-actin dynamics have also been observed during HSV-1 neuronal infection, which is mediated by the activation level of Cofilin1 (41). Moreover, Japanese encephalitis virus entry triggers a biphasic GTP-bound level of Rac1 and RhoA, accompanied by actin cytoskeleton remodeling (39).

Rac1, RhoA, and Cdc42 were the first identified and best characterized Rho-GTPases (16). We initially measured the endogenous levels of Rac1, RhoA, and Cdc42 and found no significant change during RABV infection (Fig. 2). Inactive Rac1 suppressed viral infection, which was validated by NSC23766 treatment and negative-dominant Rac1 transfection (Fig. 3). However, as for Cdc42 and RhoA, negative dominant constructs and effective inhibitors had negligible impact on RABV (Fig. 2). The inhibitory effect of IPA-3 on RABV infection was abolished upon simultaneous pretreatment with NSC23766 (Fig. 3), indicating that Rac1 exerted its role through the downstream molecule Pak1. After verifying the crucial function of Rac1 in RABV infection, we first identified Rac1 to be a cellular partner of the RABV P protein. The P-Rac1 interaction was confirmed by coimmunoprecipitation, and colocalization was observed by immunofluorescent staining of infected cells (Fig. 4). The binding region of P was located at amino 82–137. RABV P is a cofactor of RNA polymerase in viral replication and antagonizes the host’s innate immune system by exploiting cellular molecules. The 30 terminal residues (268–297 aa) of RABV P have been shown to bind STAT1/STAT3, thereby counteracting JAK-STAT and Gp130 receptor signal transduction pathways and facilitating the counteraction to the host antiviral response (42, 43). The direct interaction between the RABV P 222–297 aa sequence and the interferon-induced promyelocytic leukemia protein promotes viral infection (44). The association of the 106–131 aa sequence with focal adhesion kinase is involved in viral replication (45). Based on the P-Rac1 interaction, RABV infection and RABV P overexpression both downregulate Rac1 activity (Fig. 5). Knockdown expression of the P protein in RABV-infected cells abrogated the inhibitory impact on virus-induced Rac1 activity and downstream signaling (Fig. 5), which illustrates that RABV P is responsible for the effects of RABV infection on the actin cytoskeleton. Interestingly, we discovered that the interaction of RABV P and Rac1 T17N appeared to be negligible (Fig. 4), which indicated that the N-terminus of Rac1 played a potential role. Unfortunately, we did not specify the precise site that determines the interaction of Rac1 with RABV P. The removal of the Rac1 N-terminus (1–40 aa) did not totally abolish but did impair the interaction (data not shown). The detailed mechanism by which RABV P binding downregulates Rac1 activity remains to be further identified. Rho-GTPase activity depends on the switch between GTP and GDP, during which guanine nucleotide exchange factors (GEFs) catalyze the exchange of GDP for GTP while GTPase-activating proteins (GAPs) increase intrinsic GTP hydrolysis (46). The GEFs and GAPs involved remain to be further elucidated. To determine whether the interaction between RABV P and Rac1 exerts an influence on GTP and GDP switching, further exploration is needed. A series of posttranslational modifications, such as lipidation, ubiquitination, phosphorylation, and adenylation, regulate Rac1 activity (47). Multiple ubiquitin ligases have been documented to target Rac1 for degradation (48, 49). For instance, E3 ubiquitin ligase HACE1 downregulates Rac1 activity and functions as a suppressor in Rac1-mediated tumor progression (50, 51). IAV NS1 protein directly binds to Rac1 and modulates its activity as well as signal transduction mediated through ubiquitination and SUMOylation (23). Whether there is any post-translational modification enzyme involved in RABV-induced Rac1 activity regulation and whether the interacting region overlaps with that of RABV P and Rac1 needs further investigation.

RABV occupies the CNS as a reservoir for replication and provokes a storm of abnormal neurological symptoms (3). In contrast to robust cell loss, RABV generates severe destruction and disorganization of neuronal processes (6, 7). Neurological clinical symptoms, including severe agitation, depression, hydrophobia, paralysis, and neurocognitive impairments, successively emerge throughout the whole pathological course of the disease (3). It has long been hypothesized that fatal rabies may result from neuronal dysfunction, not neuronal damage. However, the detailed mechanism by which RABV infection causes neurological symptoms and damage remains in question. The actin cytoskeleton plays a role in supporting neurite shape and synaptic plasticity (52, 53), and a broken or discontinued cytoskeleton leads to abnormal nervous symptoms and induces neurodegenerative diseases such as Parkinson’s disease and Alzheimer’s disease (54–56). In this work, we observed the dissolution of actin stress fibers following RABV infection (Fig. 6), which is consistent with previous reports showing that RABV causes actin bundle decrease and dendritic injuries *in vivo* (10, 11). The disruption of cytoskeletal integrity may provide a possible explanation for the degeneration of neuronal processes caused by RABV infection. Although how rabies induces aberrant neuronal processes remains unclear, ameliorating neurological disorders should be the aim of therapeutic development.

Overall, our study provides the first evidence showing that Rac1 associates with RABV infection and discloses the mechanism by which RABV triggers biphasic neuronal cytoskeleton rearrangement. These findings may lead to a better understanding of RABV neuropathology and suggest a potential new avenue for therapy development.

## MATERIALS AND METHODS

### Cell culture, virus, and infections

N2a (CCL-131, ATCC) and 293T cells (ACS-4500, ATCC) were cultured in Dulbecco’s modified Eagle medium (DMEM; D5796, Sigma) supplemented with 10% fetal bovine serum (A6806–15, NQBB), 100 µg/mL of penicillin, and 100 µg/mL of streptomycin (P1400, Solarbio). Cells were maintained at 37°C and 5% CO_2_ in a humidified incubator. The CVS-11 was kindly given to us by the Changchun Veterinary Research Institute, OIE Rabies Reference Laboratory. The virus was propagated in N2a cells. To generate virus stocks, N2a cells were grown in monolayers of T25 flask at 70% confluence and infected with CVS-11 at MOI of 0.1, then harvested after at 72 h p.i. The supernatant containing viruses was collected by centrifugation following three freeze-thaw cycles and stored at −80°C. Viral titer was determined by calculating the TCID50 on N2a cells using the Karber method. N2a cells were inoculated with CVS-11 at indicated MOIs. Viral adsorption to cells was performed at 4°C to achieve infection synchronization, followed by three washes with cold phosphate-buffered saline (PBS). Subsequently, the cells were shifted to 37°C to allow internalization for the indicated time.

### Pharmacological inhibitors and antibodies

Pharmacological inhibitors were dissolved in dimethyl sulfoxide (DMSO; V900090, Sigma) or DMEM medium following the manufacturer’s recommendation and used at indicated concentration. Latrunculin A (Lat A; ab144290, Abcam), Jasplakinolide (Jasp; 141409, Abcam), CCG-1423(S7719, Selleck), ML141(HY-12755, MCE), AG-1478 (HY-13524, MCE), Wortmannin (HY-10197, MCE), AKT VIII (HY-10355, MCE), NSC23766 (S8031, Selleck), and IPA-3 (HY-15663, MCE) were employed. Antibodies used are as follows: Fluorescein isothiocyanate anti-Rabies Monoclonal globulin antibody (800–092, FUJIREBIO), anti-Flag (8146S, Cell Signaling Technology), anti-Rabies Virus (5B12; NB110–7542, Novus), anti-RhoA (2117S, Cell Signaling Technology), anti-Cdc42 (2462S, Cell Signaling Technology), anti-Rac1 (ab282581, Abcam), anti-GFP(66002–1-lg, Proteintech), anti-EGFR (4267S, Cell Signaling Technology), anti-p-EGFR (3777S, Cell Signaling Technology), anti-PI3K (66225–1-lg, Proteintech), anti-p-PI3K (17366S, Cell Signaling Technology), anti-Akt (10176–2-AP, Proteintech), anti-p-Akt (66444–1-lg, Proteintech), anti-Pak1 (2601S, Cell Signaling Technology), anti-p-Pak1 (D155068, BBI LIFE SCIENCES), anti-Limk1 (3842S, Cell Signaling Technology), anti-p-Limk1 (YP0591, IMMUNOWAY), anti-Cofilin1 (ab428424, Abcam), anti-p-Cofilin1 (3313S, Cell Signaling Technology), anti-GAPDH (1A6; MB001, Bioworld), horseradish peroxidase (HRP)-labeled goat anti-mouse antibody (AP308P, Sigma), and HRP-conjugated goat anti-Rabbit IgG (SA00001-2, Proteintech). The antibody against RABV P and N were prepared in our lab.

### Plasmids, siRNA, and transfection

The dominant-negative (DN, T19N, #12967) and wild-type (WT, #12965) RhoA, the dominant-negative (DN, T17N, #12976) and wild-type (WT, #12975) Cdc42, and the constitutively-active (CA, Q61L, #13720), dominant-negative (DN, T19N, #13721), and wild-type (WT, #13719) Rac1 plasmids with EGFP-conjugated at N-terminal were purchased from Addgene. Flag-tagged versions of truncated forms of RABV P, including P_19-297_ (amino acids 19–297), P_52-297_ (amino acids 52–297), P_82-297_ (amino acids 82–297), P_138-297_ (amino acids 138–297), and P_172-297_ (amino acids 172–297), were constructed and stored at our lab. siRNA of Rac1 used was purchased from Sangon Biotech and listed below: NC (5′-UUCUUCGAACGUGUCACGU-3′); siRNA-1 (5′-CGAGGACUCAAGACAGUGUUU-3′); siRNA-2 (5’- CGCAGACAGACGUGUUCUUAA-3′). RABV P’s siRNA comes from Gene Pharma: NC (5′-UUCUCCGAACGUGUCACGUTT-3′); siRNA-1 (5’- GGCCGAAGAGACUGUUGAUTT-3′); siRNA-2 (5’- CGAUAAUGUUGGAGUCCAATT-3′).

Cells were transfected with constructed plasmids using Lipofectamine LTX transfection and Plus Reagent (15338–100, Invitrogen) according to the manufacturer’s instructions. For siRNA analysis, N2a cells were seeded onto six-well plates and transfected with 25 pmol siRNA using Lipofectamine RNAiMAX Reagent (13778150, Invitrogen) according to the manufacturer’s instructions.

### MTT assay

Potential cytotoxic effects of drugs on N2a cells were evaluated with MTT reagent (M5655, Sigma). Subconfluent cells were cultured in 96-well plates and were incubated with various concentrations of pharmacological inhibitors. After cells were incubated with the indicated drug for 24 h at 37°C, 10 µL of the MTT (5 mg/mL) reagent was added for another 4 h. DMSO was subsequently added after the supernatant was extracted. The absorbance at the wavelength of 490 nm was measured by using a microplate reader (Tecan).

### Real-time qPCR analysis

Total cellular RNA was extracted using Trizol reagent (9108, TaKaRa) and processed reverse transcription PCR to synthesize cDNA using All-In-One RT MasterMix (G592, Applied Biological Materials) according to the manufacturer’s instructions. RT-qPCR was performed using FastStart SYBR green real-time PCR Master Mix (04913914001, Roche) on the 7,500 real-time PCR system (Applied Biosystems). The cycling conditions were as follows: 40 cycles for 95°C 10 min, 95°C 15 s, and 60°C 1 min. The RT-qPCR primer sequences are as follows: RABV N Forward primer (TCAAGAATATGAGGCGGCTG) and RABV N Reverse primer (TGGACGGGCTTGATGATTGG); RABV P Forward primer (CAACCTTGGTGAGATGGTTAGGGT) and RABV P Reverse primer (GTTGACCGTGACATAGGATAC); RhoA Forward primer (GCCTTGAGCCTTGCATCTGA); RhoA Reverse primer (ACCTCTGGGAACTGGTCCTT); Cdc42 Forward primer (CCATCGGAATATGTACCAACTGTTT); Cdc42 Reverse primer (ACTAGAAAAACATCTGTCTGTGGAT); Rac1 Forward primer (TCTCCTACCCGCAGACAGTT) and Rac1 Reverse primer (AGGATACCACTTTGCACGGA); GAPDH Forward primer (TGTGTCCGTCGTGGATCTGA) and GAPDH Reverse primer (TTGCTGTGAAGTCGCAGGAG).

### Coimmunoprecipitation assay

Cells were lysed in IP Lysis Buffer (87787, Thermo Scientific) with Protease inhibitors added. After protein concentration measurement with bicinchoninic acid (BCA) Protein Assay kit (23227, Thermo Scientific), cell lysates were preincubated with 10 µg IgG for 2 h and 20 µL of protein A/G Plus-agarose (26162, Thermo Scientific) at 4°C for 1 h. Then, supernatants were added with 15 µg immunoprecipitating antibody at 4°C overnight and conjugated to 30 µL Protein A/G Agarose Beads at 4°C for 4 h. After washed with IP Lysis Buffer for three times, precipitated protein complexes were eluted and analyzed by western blot.

### Immunofluorescence analysis

Cells were grown to 60% confluence in a 24-well plate in which glass coverslips were seeded. At indicated time postinfection or posttransfection, cells were fixed with 4% paraformaldehyde for 30 min at room temperature and permeabilized with 0.1% Triton X-100 for 10 min. After blocked with 5% goat serum for 2 h, the cells were incubated with primary antibodies for 2 h at 37°C and fluorochrome-conjugated secondary antibodies for 1 h. The cell nucleus was stained with Hoechst (C0021, Solarbio; 1:1,000). The antibodies and dilution rate were listed as follows: anti-Flag (8146S, Cell Signaling Technology; 1:1,600), anti-Rac1 (ab282581, Abcam; 1:50), and Alexa Fluor 594 goat anti-mouse IgG (AB_2534116, Invitrogen; 1:500). Actin was stained with fluorescein isothiocyanate Alexa Fluor-488 phalloidin (A12379, Invitrogen; 1:1,000). Fluorescence images were acquired using Olympus FV3000 confocal laser scanning microscope (Olympus, Japan), and the fluorescence intensity was processed and analyzed using ImageJ software.

### Rac1 activation assays

N2a cells were serum starved for 4 h and were infected with CVS-11 at indicated MOI or transfected with plasmids as described. At indicated times postinfection, cells were harvested and were washed three times with cold PBS at 4°C. Rac1 activation was measured using the G-LISA activation kit (BK128, Cytoskeleton) following the manufacturer’s recommendations. GTP-bound Rac1 was pulled down using Pak1-PBD-Agarose Beads (PAK02, Cytoskeleton) and detected through western blot by anti-Rac1 specific antibody and a secondary antibody conjugated to HRP. The signal of Rac1 activation in G-lisa was read by measuring absorbance at a wavelength of 490 nm using a microplate reader.

### G-Actin/F-Actin assay

G-Actin and F-Actin ratio assay were implemented using the G-Actin/F-Actin *In Vivo* Assay Kit (bk037, Cytoskeleton). After spreading the cells in 35 mm dishes for 12 h, the cells were inoculated with CVS-11 at an MOI of 10 and incubated at 4°C for 1 h before being moved to 37°C. After washing with PBS several times, 100 µL lysate was added to the dishes. The cells were harvested by thoroughly scraping with a cell scraper at each time point and incubated at 37°C for 10 min. The whole cell lysate was then centrifuged at 37°C for 5 min at 4,000 g. Following removing cell debris, the liquid supernatant was further centrifuged at 15,000 g, 37°C for 1 h, at this time, G-actin being dissolved in the supernatant; and F-actin being precipitated. Subsequently, 100 µL of F-actin depolymerization buffer was added to the F-actin pellet and held on ice for 1 h to promote actin depolymerization. The pellet was resuspended by pipetting up and down several times every 15 min to help pellet resuspension. G-actin and F-actin were mixed with SDS sample buffer and stored at −20°C for further western blot analysis.

### Western blot analysis

N2a and 293T cells were collected and lysed in RIPA Buffer (9806, Cell Signaling Technology) with phosphatase inhibitors cocktail (C500017, Sango Biotech) and proteinase inhibitors (GRF101, EpiZyme) added. After protein concentration measurement, the protein lysates were boiled with 5 × SDS PAGE buffer (P1040, Solarbio), separated using 10%–15% SDS PAGE, and transferred to a polyvinylidene fluoride membrane of 0.45 µm pore size (03010040001, Roche). Membranes were saturated for 2 h in 5% non-fat dried milk diluted in Tris buffered saline with Tween (TBST) solution and then were incubated with specific primary antibodies overnight at 4°C followed by incubation with secondary antibody for 2 h at room temperature. In the interval, the membranes were washed four times with TBST washing buffer for 15 min in interval in order to remove excessive antibodies. Chemiluminescent HRP substrate (32209, Thermo Scientific) was used as the detection substrate for the final acquisition of the images. Immunoreactive bands were visualized by the Tanon 5200 Multi Chemiluminescent Imaging System and quantified by ImageJ software. The protein intensity was normalized to GAPDH and was calculated as the fold increase relative to the control.

### Statistical analysis

Each experiment was repeated three times. The results are presented as the mean ± SDs of the mean. Statistical analysis was performed using Student’s *t* test with GraphPad Prism 9 software. A *P* value < 0.05 indicated a statistically significant difference.

## Data Availability

The data that support the findings of this study are available on request from the corresponding author.

## SUPPLEMENTAL MATERIAL

Table S1 jvi.00606-24-s0001.xlsx Proteins immunoprecipitated with Flag after N2a cells were transfected with Flag vector.

Table S2 jvi.00606-24-s0002.xlsx Proteins immunoprecipitated with Flag-P after N2a cells were transfected with Flag-P.

ASM does not own the copyrights to Supplemental Material that may be linked to, or accessed through, an article. The authors have granted ASM a non-exclusive, world-wide license to publish the Supplemental Material files. Please contact the corresponding author directly for reuse.
